# A comparative study of ethanol production using dilute acid, ionic liquid and AFEX™ pretreated corn stover

**DOI:** 10.1186/1754-6834-7-72

**Published:** 2014-05-13

**Authors:** Nirmal Uppugundla, Leonardo da Costa Sousa, Shishir PS Chundawat, Xiurong Yu, Blake Simmons, Seema Singh, Xiadi Gao, Rajeev Kumar, Charles E Wyman, Bruce E Dale, Venkatesh Balan

**Affiliations:** 1 Department of Chemical Engineering and Materials Science, Department of Energy (DOE) Great Lakes Bioenergy Research Center (GLBRC), Michigan State University, East Lansing, MI 48824, USA; 2 Department of Biochemistry, Department of Energy (DOE) Great Lakes Bioenergy Research Center (GLBRC), University of Wisconsin, Madison, WI 53706, USA; 3 Jilin TuoPai Agriculture Products Development Ltd, Jilin, China; 4 Deconstruction Division, Joint BioEnergy Institute (JBEI), Emeryville, CA 94608, USA; 5 Biological and Material Science Center, Sandia National Laboratories, Livermore, CA 94550, USA; 6 BioEnergy Science Center (BESC), Oak Ridge National Laboratory, Oak Ridge, TN 37831, USA; 7 Department of Chemical and Environmental Engineering, Bourns College of Engineering, University of California Riverside, Riverside, CA 92507, USA; 8 Center for Environmental Research and Technology (CE-CERT), Bourns College of Engineering, University of California Riverside, 1084 Columbia Avenue, Riverside, CA 92507, USA

**Keywords:** AFEX, Dilute acid, Ionic liquid, Pretreatment, Enzymatic hydrolysis, Cellulosic ethanol

## Abstract

**Background:**

In a biorefinery producing cellulosic biofuels, biomass pretreatment will significantly influence the efficacy of enzymatic hydrolysis and microbial fermentation. Comparison of different biomass pretreatment techniques by studying the impact of pretreatment on downstream operations at industrially relevant conditions and performing comprehensive mass balances will help focus attention on necessary process improvements, and thereby help reduce the cost of biofuel production.

**Results:**

An on-going collaboration between the three US Department of Energy (DOE) funded bioenergy research centers (Great Lakes Bioenergy Research Center (GLBRC), Joint BioEnergy Institute (JBEI) and BioEnergy Science Center (BESC)) has given us a unique opportunity to compare the performance of three pretreatment processes, notably dilute acid (DA), ionic liquid (IL) and ammonia fiber expansion (AFEX^TM^), using the same source of corn stover. Separate hydrolysis and fermentation (SHF) was carried out using various combinations of commercially available enzymes and engineered yeast (*Saccharomyces cerevisiae* 424A) strain. The optimal commercial enzyme combination (Ctec2: Htec2: Multifect Pectinase, percentage total protein loading basis) was evaluated for each pretreatment with a microplate-based assay using milled pretreated solids at 0.2% glucan loading and 15 mg total protein loading/g of glucan. The best enzyme combinations were 67:33:0 for DA, 39:33:28 for IL and 67:17:17 for AFEX. The amounts of sugar (kg) (glucose: xylose: total gluco- and xylo-oligomers) per 100 kg of untreated corn stover produced after 72 hours of 6% glucan loading enzymatic hydrolysis were: DA (25:2:2), IL (31:15:2) and AFEX (26:13:7). Additionally, the amounts of ethanol (kg) produced per 100 kg of untreated corn stover and the respective ethanol metabolic yield (%) achieved with exogenous nutrient supplemented fermentations were: DA (14.0, 92.0%), IL (21.2, 93.0%) and AFEX (20.5, 95.0%), respectively. The reason for lower ethanol yield for DA is because most of the xylose produced during the pretreatment was removed and not converted to ethanol during fermentation.

**Conclusions:**

Compositional analysis of the pretreated biomass solids showed no significant change in composition for AFEX treated corn stover, while about 85% of hemicellulose was solubilized after DA pretreatment, and about 90% of lignin was removed after IL pretreatment. As expected, the optimal commercial enzyme combination was different for the solids prepared by different pretreatment technologies. Due to loss of nutrients during the pretreatment and washing steps, DA and IL pretreated hydrolysates required exogenous nutrient supplementation to ferment glucose and xylose efficiently, while AFEX pretreated hydrolysate did not require nutrient supplementation.

## Background

Crude oil is the primary feedstock source for producing transportation fuels, industrial chemicals and polymers. Rising crude oil prices, political/social tensions in major oil-producing nations, and greenhouse gas emissions driving climate change have triggered worldwide research towards the development of alternative, sustainable sources of energy [1]. Lignocellulosic biofuels are recognized as a potential alternative to fossil fuels. Considerable research has been conducted in recent years to develop efficient technologies to convert plant-derived polysaccharides to ethanol [2]. Unlike corn grain-based ethanol, where the starch can be readily hydrolyzed to fermentable sugars using enzymes, the production of lignocellulosic ethanol is limited by biomass recalcitrance. Biomass recalcitrance is thought to arise largely due to the complex intertwined network of cellulose, hemicellulose and lignin that restricts enzyme accessibility [3,4]. Numerous pretreatment processes have been developed to overcome biomass recalcitrance, such as: steam explosion, hot water, dilute acid (DA), lime, phosphoric acid, ammonia (for example, ammonia fiber expansion (AFEX™), soaking in aqueous ammonia (SAA) and ammonia recycled percolation (ARP)) and ionic liquid (IL)-based pretreatments [5,6]. However, most pretreatments pose various challenges in terms of costs incurred by use of excess water, expensive chemicals and chemical recovery, feedstock handling, energy requirements and downstream processing [7].

DA, IL and AFEX pretreatments are part of the core research programs studied at the three US Department of Energy (DOE) funded bioenergy research centers, namely: Great Lakes Bioenergy Research Center (GLBRC), Joint Bioenergy Institute (JBEI) and the BioEnergy Science Center (BESC). DA (acidic) and IL (acidic or alkaline) are dry to wet product pretreatment processes, whereas AFEX (alkaline) is a dry biomass to dry product process. Considerable research has been done to streamline these processes in order to make them cost effective. Though these pretreatments still face various challenges, they can efficiently pretreat lignocellulosic biomass to aid higher sugar release during enzyme hydrolysis.

In our previous research, developed under the Consortium for Applied Fundamentals and Innovation (CAFI) I, II and III programs [8,9], we compared the performance of several pretreatment technologies. In these studies, the performance of AFEX and DA pretreatments was compared for different types of feedstock (corn stover (CS), poplar and switchgrass) [6,9,10]. However, IL pretreatment was not a part of such comparative studies. In this work, we take into consideration the fact that, after being exposed to different pretreatment methods, the resulting biomass materials do not necessarily have the same enzyme requirements (for example, greater hemicellulase requirement for AFEX versus DA treated corn stover) to achieve maximum yields. Similarly, enzymatic hydrolysates derived from the various pretreatment methods have different nutrient requirements to maximize ethanol yields. Thus, by studying downstream processing conditions that maximize product yields, we can better compare the potential of each pretreatment method on a level playing field (for example, enzyme loading, glucan loading, residence time, and so on). We carried out separate hydrolysis and fermentation (SHF) and compared the performance of corn stover solids prepared by DA, IL and AFEX pretreatments. To achieve this, we first optimized the commercial enzyme cocktails for each pretreated biomass prior to subsequent high-solids loading saccharification and fermentation. The fermentability of pretreated corn stover hydrolysates was evaluated using a recombinant yeast strain of *Saccharomyces cerevisiae* 424-A (LNH-ST) (with and without external nutrient supplementation) [11]. Material balances around pretreatment, hydrolysis and fermentation were developed to determine the fates of key biomass components (cellulose, hemicellulose and lignin) and highlight the differences in sugar and ethanol yields for the three pretreatment methodologies at industrially relevant saccharification/fermentation conditions.

## Results and discussion

### Composition of AFEX, DA and IL pretreated biomass

Conditions and parameters for DA, IL and AFEX pretreatments are summarized in Table 1. As expected, all three pretreatments exhibit distinct chemistries, as demonstrated by differences in cell wall composition among pretreated materials (see Table 2). For example, the alkaline AFEX process cleaves most of the ester linkages present in the plant cell wall [12], as confirmed by the absence of acetyl content in the AFEX pretreated corn stover (Table 2). Almost all the acetyl groups are converted to acetic acids and acetamide due to the corresponding hydrolysis or ammonolysis reactions during AFEX pretreatment. In this process, lignin and hemicellulose are partially solubilized and relocated to the biomass surface during pretreatment, leaving behind a highly porous cell wall that helps to improve enzyme access to the embedded cellulose and hemicellulose [13]. AFEX pretreatment is a dry to dry process (material enters the process dry and also leaves the process in the dry state) during which minimal carbohydrate degradation takes place [12] and negligible modifications in total polysaccharide composition are seen compared to untreated biomass (see Table 2). The reduced acid insoluble or Klason lignin (approximately 30%) levels observed after AFEX pretreatment may result from currently unknown chemical modifications at the lignin level that improve lignin solubility during sample preparation (that is, extraction with hot water and ethanol to remove interfering extractives prior to sulfuric acid hydrolysis) prior to composition analysis based on current National Renewable Energy Laboratory (NREL) laboratory analytical procedure (LAP).

**Table 1:** Pretreatment conditions used to pretreat biomass using different methods

**Method**	**Pretreatment**						**Post-wash**	
	**Temperature (°C)**	**Residence time (minutes)**	**Catalyst type**	**Catalyst loading (kg)**^**a**^	**Catalyst recyclable?**	**Water loading (kg)**^**b**^	**Post-wash water use (kg)**^**b**^	**Water temperature (°C)**
DA	160	20	Sulfuric acid	4.5	No	895.5	1,000	Room temperature
IL	140	180	IL [C2mim][OAc]	900	Yes	N/R	10,000	Room temperature
AFEX	140	15	Anhydrous ammonia	100	Yes	60	N/R	-

^a^Catalyst loading: kg/100 kg dry biomass; ^b^water use: L/100 kg dry biomass. AFEX, ammonia fiber expansion; [C2mim][OAc], 1-ethyl-3-methylimidazolium acetate; DA, dilute acid; IL, ionic liquid; N/R, not required.

**Table 2:** Compositional analysis (% wt/wt, dry weight basis) for untreated, AFEX, DA and IL treated MSU corn stover

**Composition**	**Untreated**	**AFEX**	**DA**	**IL**
Glucan	33.4 ± 3.2	33.5 ± 0.5	59.1 ± 3.0	46.9 ± 1.9
Xylan	24.9 ± 2.0	24.8 ± 0.9	6.5 ± 0.1	29.8 ± 0.5
Arabinan	3.7 ± 0.5	3.3 ± 0.4	3.6 ± 0.1	0.3 ± 0.0
Acetyl	2.1 ± 0.2	0.0 ± 0.0	0.6 ± 0.6	1.5 ± 0.1
Acid insoluble lignin^a^	17.2 ± 0.6	12.2 ± 0.2	22.2 ± 0.2	2.7 ± 0.5
Ash	3.6 ± 0.0	4.4 ± 0.3	2.5 ± 0.0	1.3 ± 0.26
Extractives	10.4 ± 0.4	24.8 ± 0.8	15.4 ± 0.8	13.1 ± 2.0

All experiments were carried out in triplicates with averages and standard deviations reported here.

^a^The acid insoluble or Klason lignin analysis method was modified to use 47 mm, 0.22 μm pore size mixed cellulose ester filter disks (Millipore Corporation, Bedford, MA, USA) during the filtration process instead of the fritted crucibles. The filtered lignin residues were dried overnight in a desiccator prior to weighing. AFEX does not remove any particular components during pretreatment. Ammonia is evaporated and all the ammonia soluble biomass components condense on the surface of the biomass [13]. However, some reactions do occur to lignin during AFEX, transforming Klason lignin (acid insoluble) to acid soluble lignin, which was not quantified in this study. AFEX, ammonia fiber expansion; DA, dilute acid; IL, ionic liquid; MSU, Michigan State University.

On the other hand, DA pretreatment cleaves not only ester linkages, but also some ether linkages present in lignin. Use of dilute sulfuric acid favors the chemical conversion of xylan to xylose, which can be further dehydrated to furfural under certain conditions [14]. These hydrolyzed sugars and their respective decomposition products are soluble in the acidic pretreatment liquor, which are hence separated from the solids after pretreatment. Subsequent washing steps (mostly to remove residual acid) aid in further removal of these soluble components from the pretreated residual solids. Therefore, these solids have a reduced xylan content compared to untreated corn stover. As a result, the percentage of glucan and lignin in the pretreated solids increased by 26% and 5%, respectively. The acetyl content decreased from 2.1% to 0.6%, possibly due to incomplete hydrolysis of ester linkages under these pretreatment conditions.

ILs, such as 1-ethyl-3-methylimidazolium acetate ([C2mim][OAc]), can solubilize plant cell wall components, which can then be selectively precipitated from solution by adding an anti-solvent (for example, water and ethanol), leaving most of the lignin behind in solution, as carried out in this study. The composition of the solids after precipitation and washing (Table 2) clearly shows an enriched fraction of glucan (46.9%) and xylan (29.8%), with only 2.7% lignin content. The residual acetyl content of IL treated corn stover is higher than that of DA and AFEX treated corn stover. This suggests that IL pretreatment may not effectively cleave acetyl residues. Since acetyl ester residues remain intact after IL treatment, it is likely that most of the ferulate ester crosslinks that are responsible for the lignin-carbohydrate complexes (LCCs) are intact after IL pretreatment. Previous work has suggested that [C_2_mim][OAc]-based pretreatment deacetylated the xylan backbone, while simultaneously acetylating the lignin from *Eucalyptus globulus*, and partially cleaving the β-ether linkages from lignin [15]. Based on this observation, it may be that the acetyl content of the IL pretreated solids might have derived from the residual acetylated lignin. Detailed work is required to further characterize the acetylated components of IL pretreated cell walls and the role of [C2mim][OAc] in acetylation reactions during IL pretreatment of corn stover.

### Enzyme optimization

**Figure 1:**
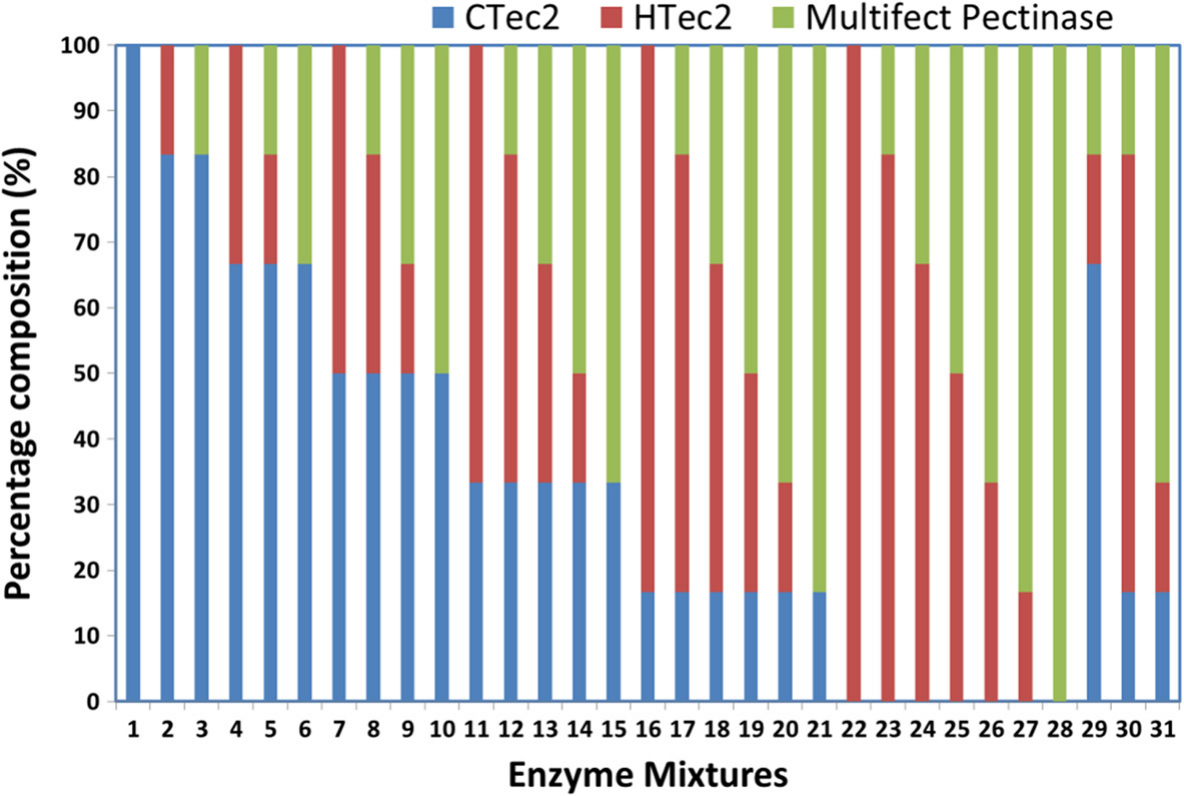
**Three commercial enzyme combinations (based on total protein loading) used for optimizing enzyme cocktail for DA, IL and AFEX biomass.** AFEX, ammonia fiber expansion; DA, dilute acid; IL, ionic liquid.

**Figure 2:**
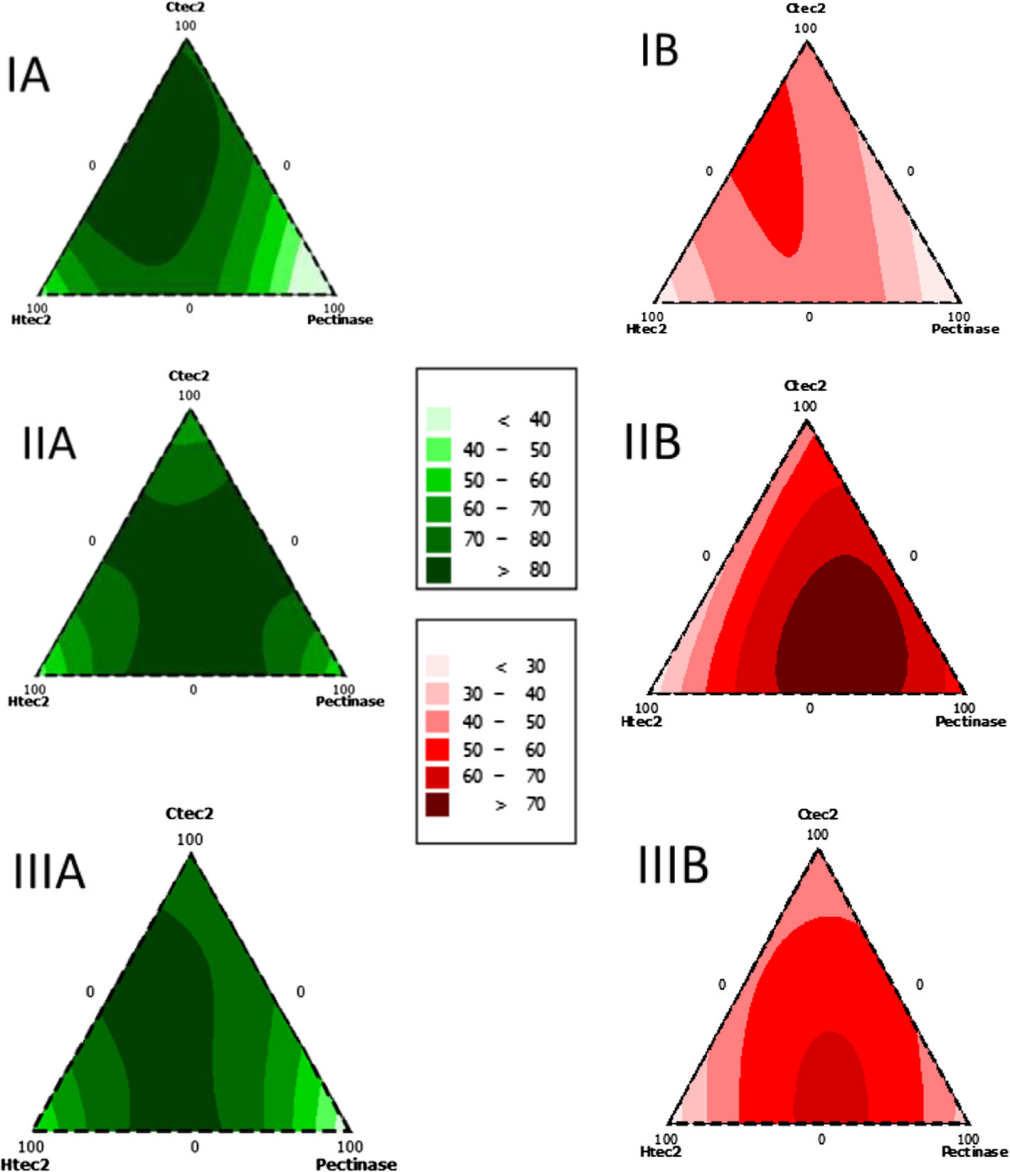
**Optimum commercial enzyme mixture ratio-based Minitab model predicted contour plots for ternary combinations of CTec2, HTec2 and Multifect Pectinase. (I)** DA, **(II)** IL and **(III)** AFEX pretreated corn stover at 0.2% glucan loading. **(A)** Glucan conversions are shown on the left and **(B)** xylan conversions are shown on the right. The total protein loading was 15 mg protein/g glucan. AFEX, ammonia fiber expansion; DA, dilute acid; IL, ionic liquid.

Commercial enzyme cocktail mixtures were optimized for DA, IL and AFEX treated corn stover to maximize saccharification yields for each pretreated substrate. It is reasonable to assume that corn stover subjected to different pretreatment methodologies will require a unique cocktail of enzymes to achieve optimum conversion. For example, DA pretreatment removes most of the xylan from corn stover and should therefore require lower xylanase activity when compared to AFEX and IL treated corn stover. To account for these differences in cell wall composition, enzyme cocktails were optimized for each pretreated substrate.The design of experiments was carried out using Minitab® software (Coventry, UK), utilizing 31 unique enzyme combinations, composed of various ratios of three commercial enzymes: CTec2 (cellulase), HTec2 (hemicellulase) and Multifect Pectinase (accessory) (Figure 1). The enzymatic hydrolysis was performed in a 96-well microplate at 0.2 wt% glucan loading and total enzyme protein loading of 15 mg/g glucan with the relative protein ratios varying from 0 to 100% of the total added protein. The performance of different enzyme combinations with respect to glucan and xylan conversions for each pretreatment is shown in Figure 2. The results demonstrate that for maximum polysaccharide conversion, DA-CS requires 67% Ctec2 and 33% Htec2 enzymes, IL-CS requires all three enzymes in similar proportions, whereas AFEX-CS requires 66.7% Ctec2, 16.7% Htec2 and 16.7% Multifect Pectinase.

It is not surprising that DA-CS requires no additional accessory enzymes and only requires 33% hemicellulase (Htec2) due to the lower levels of xylan (6.5%) and arabinan (3.6%). Similarly, 67% cellulase (Ctec2) in the optimized cocktail for DA-CS correlates with the higher glucan content, which represents approximately 85% of the total carbohydrates in the DA pretreated solids.

On the other hand, AFEX and IL pretreated corn stover contains more hemicelluloses, and thus required 16.7% and 33%, respectively, of each accessory enzyme. Even though the ratio of glucan to xylan is higher in IL-CS as compared to AFEX-CS, it requires a lower ratio of cellulase to hemicellulase. This observation correlates with the fact that AFEX-CS has higher lignin content (approximately 4.5 fold) than IL-CS. Lignin content in biomass is known to inhibit cellulases more readily than hemicellulases [16]. Moreover, IL-CS is known to decrystallize cellulose and partially convert native crystalline cellulose I to highly amorphous cellulose II [17]. Decrystallized cellulose is more amenable to enzymatic hydrolysis than the native cellulose, thereby requiring relatively less cellulase in the enzyme mixture. Thus, from our results, optimal enzyme mixtures correlate well with the content of cellulose, hemicellulose and lignin, as well as the crystalline state of cellulose. Other factors influencing these results may include the relative influence of non-native decomposition products formed during pretreatment on enzyme activity [18,19].

### Enzymatic conversion of biomass to sugars at high solid loading

**Figure 3:**
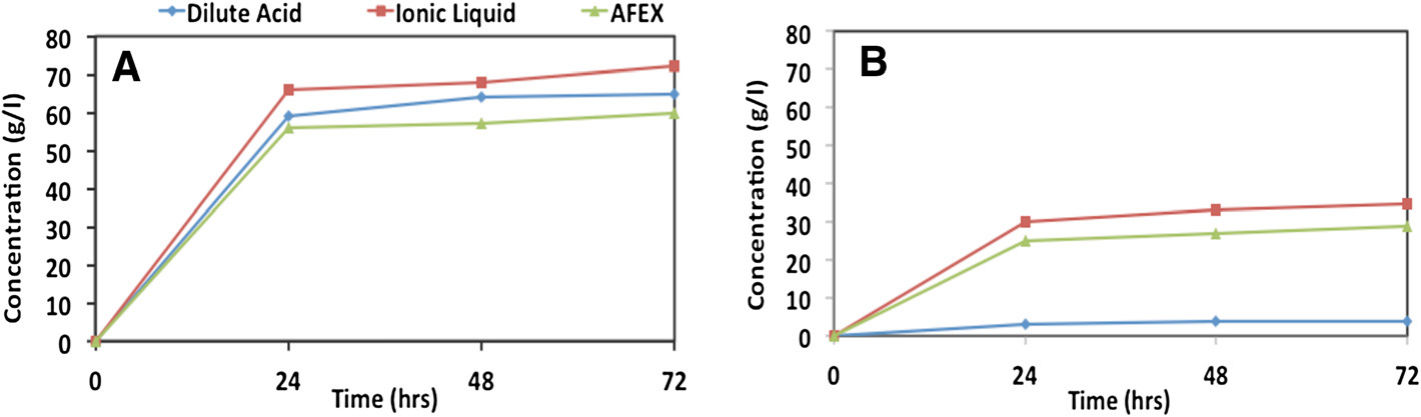
**Time course profile of glucose and xylose concentrations during high solid loading hydrolysis of pretreated corn stover. (A)** Glucose and **(B)** xylose. AFEX, ammonia fiber expansion.

The pretreated substrates were enzymatically hydrolyzed at high solid loading (6% glucan loading) in a fermenter, using the respective optimized enzyme mixtures, operating at 50°C with pH controlled at 4.8. As mentioned previously, these optimized enzyme mixtures were determined by 0.2% glucan loading enzymatic hydrolysis, performed in a high-throughput microplate format. It is possible that the optimum enzyme mixtures may vary with increased solid loadings and saccharification conditions, since some of these enzymes may be more susceptible to shear stress and substrate/end product inhibition [20]. These factors may promote deactivation/inhibition of enzymes and thereby change the relative ratios of enzymes for an optimum mixture. Thus, the extrapolation of optimum enzyme mixtures to higher solid loadings represents a limitation of this study. However, this factor should not significantly impact the final conclusions of this paper and is a previously acknowledged limitation in peer-reviewed literature [21].Throughout the course of high solid loading hydrolysis of the pretreated substrates, liquid samples were taken every 24 hours to estimate monomeric sugar concentrations. Time course data for the monomeric sugar concentrations are given in Figure 3. The first 24 hours were critical for enzymatic hydrolysis, since over 50 g/L of glucose was achieved for all three pretreated substrates (Figure 3A). On the other hand, only 4 to 6 g/L of additional glucose was produced in the following 48 hours. Moreover, the decrease in the rate of enzymatic hydrolysis observed between 24 to 72 hours is not significantly different between the three pretreatments/substrates, and is approximately 4% to 5% of the rates of enzymatic hydrolysis achieved in the first 24 hours.

During high solid loading enzymatic hydrolysis, the liquid volume does not represent the total reaction volume. As the solids concentration increases (from 1% glucan loading to 6% as used in this study), the assumption that the solution volume is all liquid is increasingly incorrect as a larger fraction of the volume is occupied by solids. Therefore, it is not possible to calculate conversion at high solid loading without performing solid–liquid separations, measuring liquid volume and concentration of sugars in the liquid fraction. For this reason a thorough mass balance was performed on the hydrolysis products as described previously by Garlock *et al.*[6] to determine the final glucan and xylan conversion to mono- and oligosaccharides.

IL-CS gave higher glucan conversion after 24 hours, yielding 66 g/L of glucose and therefore achieving the highest final concentration of glucose of all three substrates after 72 hours (72 g/L, 100% glucan conversion). DA-CS produced 59 g/L of glucose in the first 24 hours and reached a maximum of 65 g/L after 72 hours (88% glucan conversion). Similarly, AFEX produced 56 g/L of glucose in 24 hours; however, since the rate of glucose release is slightly lower than that of DA-CS during the remaining 48 hours, the final concentration of glucose peaked at 60 g/L (79% glucan conversion).

These differences in glucose release rate and final glucose concentrations are a consequence of the various physicochemical properties of the differently pretreated substrates. For example, lignin content is low in IL-CS. Lignin is widely known to affect enzymatic hydrolysis by inhibiting cellulases and hemicellulases [16,22-25]. Moreover, IL pretreatment modifies cellulose structure by converting native cellulose I_β_ to cellulose II with a significantly reduced crystallinity index (CrI) [17,26-28]. Cellulose II prepared by mercerization with 25% NaOH had enhanced the performance of cellulases by 1.6 fold when compared to native cellulose I_β_[29]. However, it is likely that the amorphous cellulose that is also produced during formation of cellulose II is the primary cause for improvement in net hydrolysis rate. The other pretreatments in this study do not modify the native crystal structure of cellulose present in corn stover. DA pretreatment is known to increase cellulose CrI due to a selective decomposition of the amorphous portions of cellulose, which are more susceptible to acid degradation as suggested by Kumar *et al*. [27,30]. With respect to AFEX pretreatment, earlier reports did not observe any major effect on cellulose CrI under current AFEX conditions [26]. Even though previous observations suggest a decrease of cellulose digestibility with increasing CrI of cellulose (on pure substrate), it is not possible to address differences in enzymatic hydrolysis performance based on CrI measurements with these substrates. This is because the pretreated biomass materials are composed of different fractions of amorphous-like materials that impact not only enzymatic digestibility (for example, lignin and hemicellulose) but also affect the accuracy of CrI estimation using X-ray diffraction.

High concentrations of xylo-oligomers inhibit cellulases and could also contribute to the lower glucan conversion seen for AFEX-CS compared to DA-CS [30,31]. This hypothesis is supported by the accumulation of xylo-oligomers during enzymatic hydrolysis of AFEX-CS, which reaches 19 g/L after 72 hours of incubation.In Figure 3B, xylose concentrations of hydrolysates are presented as a function of enzymatic hydrolysis time for the various pretreated feedstocks. Since DA-CS contains only 6.5% of xylan, lower xylose concentrations (4 g/L) can be expected in the hydrolysate. This xylose concentration corresponds to 48% xylan conversion, which is relatively low when compared to the other pretreated substrates (79% for IL-CS and 52% for AFEX-CS). It is possible that the xylan present in DA-CS is more recalcitrant to enzymatic hydrolysis since it could not be hydrolyzed to soluble oligomeric and monomeric xylose during acid pretreatment.

Similar to glucan hydrolysis, most of the hydrolyzed xylan was released in the first 24 hours in pretreated corn stover with all three pretreatment processes. IL-CS produced the highest concentration of xylose among all pretreated feedstocks (35 g/L) after 72 hours, which represents 79% of the total xylan conversion. The low lignin composition of IL-CS could be an important factor contributing to high xylan conversion and reduced enzyme inhibition during enzymatic hydrolysis [16]. AFEX-CS released 29 g/L of xylose in 72 hours of enzymatic hydrolysis, representing about 52% xylan conversion. However, 78% of AFEX-CS xylan was solubilized to monomeric and oligomeric sugars. The xylo-oligomers content is much higher in AFEX (33%) than IL (11%) and DA (23%) hydrolysates. The total xylan conversions for IL and DA (that is, xylose and xylo-oligomers) reached 88% and 62%, respectively, after 72 hours. Although the yeast used in this study is able to ferment only xylose to ethanol, it is not capable of utilizing xylo-oligomers, in common with most industrial ethanologens. Therefore, ethanol yields will depend on the conversion of xylan to monomeric xylose and not the total xylan conversion, that is, xylose and xylo-oligomers.

### Ethanol fermentation

**Figure 4:**
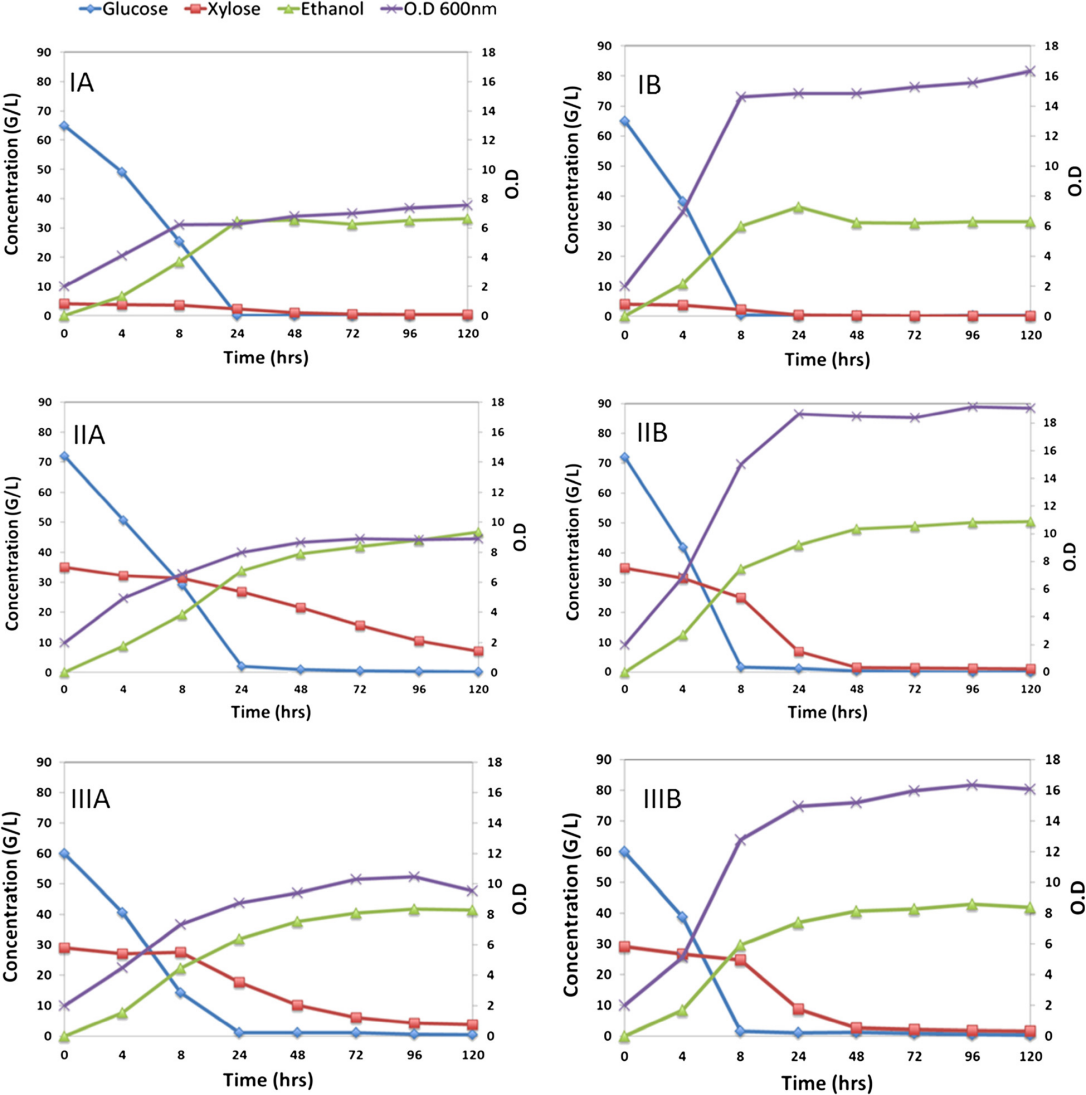
**Fermentation product and cell growth profiles for three different pretreated corn stover hydrolysates. (I)** DA, **(II)** IL and **(III)** AFEX. **(A)** Without external nutrients and **(B)** with external nutrient supplementation. AFEX, ammonia fiber expansion; DA, dilute acid; IL, ionic liquid.

Fermentation experiments were performed using the recombinant xylose-fermenting yeast strain *S. cerevisiae* 424A (LNH-ST), kindly provided by Professor Nancy Ho of Purdue University (West Lafayette, IN, USA) [32]. Fermentation product profiles for IL, AFEX and DA corn stover hydrolysates are shown in Figure 4, where glucose, xylose, ethanol and cell density (OD_600_) were monitored during 120 hours of fermentation. Since the three pretreatment methodologies considered in this work exhibit distinct mechanisms of action, the relative chemical composition of the resulting hydrolysates varies significantly [12,26,30]. In this context, since nutrient availability is one of the major factors that influences fermentation performance, we compared the fermentability of these hydrolysates both in the absence (Figure 4A) and in the presence (Figure 4B) of adequate external nutrient supplementation (yeast extract peptone and urea) required for an efficient microbial sugar metabolism [33]. The hydrolysates contain different initial concentrations of glucose and xylose, which will result in different concentrations of ethanol. Since final ethanol concentration is not an adequate metric by itself for comparing fermentation performances in this study, the data are analyzed and discussed with respect to ethanol metabolic yield, fermentation rates and percentage sugar consumption. Ethanol metabolic yield was calculated from consumed glucose and xylose during fermentation, based on the theoretical ethanol yield (0.51 g ethanol per g glucose/xylose).

In the absence of nutrient supplementation, glucose metabolism was fastest for AFEX-CS hydrolysate, with a maximum rate of 5.72 g.L^−1^.h^−1^ (Table 3). In agreement with previous reports, xylose fermentation occurred only after glucose was totally consumed by *S. cerevisiae* 424A (LNH-ST). Diauxic lag is typical of this microbial strain, which can be observed in nutrient-rich environments such as synthetic media composed of Yeast Extract Peptone (YEP), glucose and xylose [32]. The AFEX-CS hydrolysate also provided the highest xylose consumption rate (0.47 g.L^−1^.h^−1^) without additional nutrients. However, even in these optimal conditions, *S. cerevisiae* 424A (LNH-ST) was unable to completely consume xylose in all the hydrolysates within the first 120 hours of fermentation. DA-CS hydrolysate showed the highest percentage xylose consumption among the three substrates (91%), which relates to low initial concentration of xylose (4 g/L) in DA-CS hydrolysate, whereas AFEX-CS and IL-CS hydrolysates contained 29 g/L and 35 g/L of xylose, respectively.

**Table 3:** Ethanol fermentation performances of different pretreated biomass hydrolysates

**Ethanol fermentation**	**Parameter**	**DA**	**IL**	**AFEX**
**Without nutrient supplementation**	Metabolic yield (%)	93	90	98
	Glucose consumption (%)	100	100	99
	Xylose consumption (%)	91	70	84
	Maximum glucose consumption rate (g.L^−1^.h^−1^)	4.95	5.36	5.72
	Maximum xylose consumption rate (g.L^−1^.h^−1^)	0.07	0.24	0.47
**With nutrient supplementation**	Metabolic yield (%)	90	93	97
	Glucose consumption (%)	100	100	99
	Xylose consumption (%)	100	97	94
	Maximum glucose consumption rate (g.L^−1^.h^−1^)	8.09	8.80	7.30
	Maximum xylose consumption rate (g.L^−1^.h^−1^)	0.16	1.18	0.87

Metabolic yield calculated based on the theoretical ethanol yield from consumed glucose and xylose, 0.51 g ethanol/g sugar. AFEX, ammonia fiber expansion; DA, dilute acid; IL, ionic liquid.

The superior fermentation performance of the AFEX hydrolysate without nutrient supplementation when compared to IL-CS and DA-CS hydrolysates is likely due to the fact that the AFEX process requires no washing steps thereby preserving essential biomass nutrients for fermentation. Additionally, unlike IL and DA, AFEX pretreatment does not produce a highly inhibitory pretreatment mixture [12], thus avoiding the detoxification and extensive washing steps that remove nutrients from the biomass [34,35]. This hypothesis is supported by the lower cell growth rates observed in both IL-CS and DA-CS hydrolysates, when compared to AFEX-CS hydrolysate (Figure 4A), suggesting that both IL-CS and DA-CS hydrolysates were nutrient-limited for yeast cell growth. The superior fermentability of AFEX-CS hydrolysates in the absence of nutrient supplementation was also reflected by a higher metabolic yield of 98%, compared to 93% and 90% for DA-CS and IL-CS hydrolysates, respectively (Table 3).

When the hydrolysates were supplemented with nutrients, an increase in cell growth (Figure 4B) and sugar consumption rate were observed for all three hydrolysates. The highest final cell density (18.5 at OD_600_) was observed for the IL-CS hydrolysate as it contains a higher concentration of fermentable sugars (glucose and xylose) compared to the other two pretreated corn stover hydrolysates. This observation also supports the earlier hypothesis that IL-CS and DA-CS hydrolysates were nutrient-limited for *S. cerevisiae* 424A (LNH-ST) fermentation (Figure 4A). Nutrient supplementation helped the yeast to ferment most glucose in 8 hours and xylose in 48 hours (Figure 4B and Table 3). From these observations we conclude that xylose fermentation performance by *S. cerevisiae* 424A (LNH-ST) depends on nutrient availability in the media, as previously observed by Lau *et al.*[34]. Comparing the xylose fermentation rates between the hydrolysates, IL-CS hydrolysate leads with the highest rate (1.18 g.L^−1^.h^−1^), followed by AFEX-CS (0.87 g.L^−1^.h^−1^) and DA-CS hydrolysates (0.16 g.L^−1^.h^−1^). The lower xylose consumption rate observed in DA-CS hydrolysate can be attributed to its low xylose concentration (4 g/L), while the superior xylose fermentation performance of IL-CS hydrolysate demonstrates that it benefited the most from nutrient supplementation since only 70% of the xylose was fermented at a maximum rate of 0.24 g.L^−1^.h^−1^ when no nutrients were supplemented. This hypothesis is further supported by the marginal increase in metabolic yield from 90% to 93% after supplementing the IL-CS hydrolysate with nutrients, while the opposite trend was observed for the other two hydrolysates. The medium that benefited the least from nutrient supplementation was DA-CS hydrolysate, since the metabolic yield decreased by 3% (Table 3) and the final ethanol concentration did not vary significantly. This result was predictable considering that nutrient supplementation tends to benefit xylose fermentation to a greater extent than glucose fermentation and DA-CS hydrolysate contains low levels of xylose.

### Process mass balances

**Figure 5:**
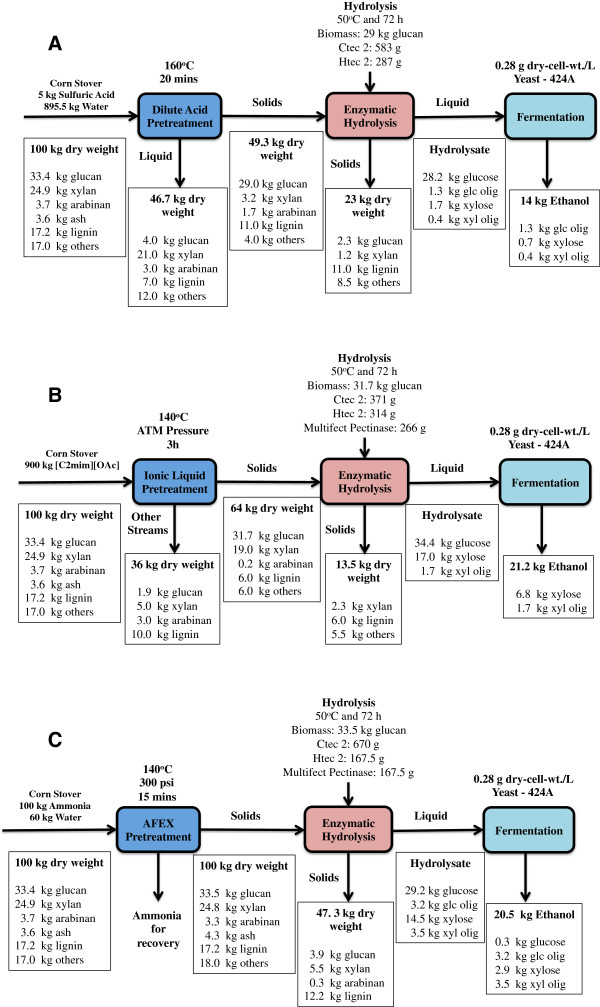
**Material balances during pretreatment, hydrolysis and fermentation for three different processes. (A)** DA-, **(B)** IL- and **(C)** AFEX-based pretreatments. AFEX, ammonia fiber expansion; DA, dilute acid; IL, ionic liquid.

The results obtained from pretreatment, enzymatic hydrolysis and fermentation were used to develop a process mass balance for each pretreatment technology (Figure 5). AFEX pretreatment utilized 100 kg of ammonia and 60 kg of water per 100 kg of dry untreated corn stover, generating a solid stream (pretreated corn stover) and a gaseous stream (mostly composed of ammonia and water vapor). The ammonia released after AFEX pretreatment can be recycled and reused in an industrial system; however, the mass balance for ammonia was not performed in this study. From previous studies, it is known that about 3 kg of ammonia left behind per 100 kg of dry untreated corn stover, mostly due to the ammonolysis and hydrolysis reactions mentioned previously [12,13]. Corn stover was essentially completely recovered after AFEX pretreatment and only minor changes were observed in its composition.

DA pretreatment utilized 4.5 kg of sulfuric acid and 895.5 kg of water per 100 kg of corn stover, followed by washing steps utilizing additional water at room temperature. In an industrial setup, after the pretreatment process, sulfuric acid is carried with the liquid stream and neutralized with an alkali (for example, CaO, CaCO_3_ or NH_4_OH). The liquid stream is rich in xylose that can be fermented to ethanol by xylose-fermenting microbes when properly conditioned and detoxified [36,37]; however, in this work this stream was not evaluated for ethanol conversion. The recovered solid stream represents about 49.3% of the untreated corn stover, primarily because of the xylan and other extractives removal during pretreatment.

In the case of IL pretreatment, 900 kg of [C_2_mim][OAc] was used per 100 kg of corn stover. The residual IL was removed from the solid biomass after pretreatment with a series of water and ethanol washes. In an industrial setup, the liquid stream can be processed to recycle and reuse the IL in subsequent pretreatment steps [38,39]; however, the mass balance over the recycling process was not performed in this study. IL pretreatment was able to remove most of the lignin from corn stover, generating a solid fraction that is enriched in glucan (46.9%) and xylan (29.8%). This washed solid fraction represents 64% of the total inlet biomass and was the stream subjected to the enzymatic hydrolysis step.

Enzymatic hydrolysis of the pretreated corn stover was performed at 6% glucan loading for 72 hours using the optimal combination of commercial enzyme cocktails determined by the previously discussed combinatorial experiments. For AFEX pretreated biomass, 78.6% of the glucan was converted to monomeric glucose during enzymatic hydrolysis, while 52% of the xylan was converted to monomeric xylose. However, the total soluble sugars present in the hydrolysate (monomeric and oligomeric) represent 88.3% and 77.7% of the initial glucan and xylan, respectively. The residual solids stream is composed of 20.5% (wt/wt) of unhydrolyzed carbohydrates and 26% (wt/wt) of lignin [40,41].

During enzymatic hydrolysis of DA-CS, it was possible to convert 87.6% and 48% of initial glucan and xylan to monomeric sugars, respectively. However, the monomeric xylose that was converted during enzymatic hydrolysis represents only 1.5 kg per 100 kg of the initial corn stover that was fed into the pretreatment operation. This is a consequence of the initial xylan solubilization during DA pretreatment. The total soluble glucan and xylan produced during enzymatic hydrolysis of DA corn stover represents 92.2% and 62.3% of the total glucan and xylan fed into the hydrolysis vessel, respectively. Similar to the AFEX pretreatment, the solid fraction leaving after enzymatic hydrolysis represents 23% of the raw biomass input and can be used to generate electrical power to supply for biorefinery operations [41].

Enzymatic hydrolysis of IL-CS was able to produce the highest glucan and xylan conversions to monomeric sugars among the three processes considered in this study. The monomeric glucose and xylose conversions during enzymatic hydrolysis were 100% and 78.9%, respectively. When compared to the other pretreated feedstocks, IL corn stover did not generate a significant amount of gluco-oligomers during enzymatic hydrolysis. However, we observed 1.7 kg of xylo-oligomers for 100 kg of corn stover fed. This corresponds to 9.4% of the xylan that was carried through enzymatic hydrolysis. More importantly, the fermentable sugar recovery (based on the initial corn stover input) for the IL-based process was the highest among the three cases considered here. The IL-based process was able to convert 79% of the initial sugars present in the untreated corn stover to fermentable sugars, while AFEX- and DA-based processes converted 67% and 46%, respectively. Potentially, the DA-based process can utilize the xylose-rich residue derived from pretreatment wash stream during fermentation. In this scenario, the sugar recovery for a DA-based process will improve about 30%, yielding 76% of total fermentable sugar recovery. However, the fermentability of this pretreatment stream was not considered in this work.

The final ethanol yields of the processes evaluated in this work are a direct consequence of the nature of the different pretreatments. The AFEX-based process was able to produce 20.5 kg of ethanol per 100 kg of biomass, while DA- and IL-based biorefineries were able to produce 14 kg and 21.2 kg, respectively. The lower ethanol value for the DA process is a consequence of the extensive xylan removal during pretreatment. Although the AFEX process presented the lowest monomeric glucose and xylose conversions during enzymatic hydrolysis, it preserved most of the sugars throughout the ethanol production process and capitalized on high ethanol metabolic yields during fermentation (Table 3). AFEX has another property that was not explored in this study. Since biomass is left dry following AFEX, it can be fed in very high concentrations, thereby achieving potentially higher ethanol concentrations, in contrast with DA and IL pretreatments. In this study, pretreated DA and IL corn stover was freeze-dried, thereby enabling the 6% glucan loadings used here. In the case of the IL process, the high monomeric glucose and xylose conversions coupled with the preservation of carbohydrates up to the fermentation stage contributed to the highest ethanol yield among the three processes. The viability of these three processes is strongly dependent on the ethanol yield. However, it should be noted that the cost of ethanol production (for example, US$/gallon of ethanol) is the most important metric to evaluate the commercial potential of these three processes. Even though this work does not present full techno-economic evaluations for the various pretreatments in a process context, it provides useful insights on different aspects of the ethanol production platforms and how they depend on the choice of pretreatment.

## Conclusions

In this work, AFEX, DA and IL pretreatments were evaluated from a biorefinery processing perspective, using industrially relevant conditions for converting corn stover to ethanol. The physicochemical differences between the pretreated substrates were acknowledged in this work by optimizing the commercial enzyme cocktails to maximize sugar yields for each individual substrate. The optimum enzyme combinations were correlated to the composition of the pretreated biomass. IL pretreated corn stover was the most readily digestible among the substrates considered in this work given the nature and composition of the substrate, and it also gave improved fermentability when supplemented with nutrients. Similar to IL-CS, DA-CS was amenable to enzymatic hydrolysis and fermentation when supplemented with adequate nutrients. In this work, the soluble sugars generated during DA pretreatment were not considered for fermentation. If we consider these soluble sugars, the DA pretreatment process could potentially recover 76% of the total sugars present in untreated corn stover. Finally, AFEX pretreatment was able to produce highly digestible substrates, conserving most of the carbohydrates during the pretreatment step. AFEX was able to produce high fermentation metabolic yield (98%) even without external nutrient supplementation. The ethanol yields calculated for DA, IL and AFEX pretreated residual solids were 14, 21.2 and 20.5 kg of ethanol per 100 kg of corn stover, respectively.

## Materials and methods

### Biomass

Corn stover harvested in September 2008, was obtained from Michigan State University (MSU) Farms (East Lansing, MI, USA). The corn hybrid used was NK 49-E3 (Syngenta, Basel, Switzerland) which is a typical corn stover hybrid used in the Great Lakes region. We refer to it as MSU corn stover. The biomass was milled to a 40 mesh size and stored at 4°C until further use.

### Enzymes

Cellic® CTec2 (138 mg protein/mL, batch number VCNI0001), a complex blend of cellulase, β-glucosidase and hemicellulase, and Cellic® HTec2 (157 mg protein/mL, batch number VHN00001) were generously provided by Novozymes (Franklinton, NC, USA). Multifect Pectinase® (72 mg protein/mL, batch number 4861295753) was a gift from DuPont Industrial Biosciences (Palo Alto, CA, USA). The protein concentrations of the enzymes were determined by estimating the protein (and subtracting the non-protein nitrogen contribution) using the Kjeldahl nitrogen analysis method (AOAC Method 2001.11, Dairy One Cooperative Inc., Ithaca, NY, USA).

### Biomass pretreatment

DA pretreatment was performed at BESC (University of California, Riverside, CA, USA) at 160°C for 20 minutes with 10% w/w solid loading and 0.5% w/w sulfuric acid using a 1 L Parr reactor with two stacked pitched blade impellers (Model 4525, Parr Instruments Company, Moline, IL, USA). It took 2 minutes for the reactor to reach 160°C and another 2 minutes to bring the biomass temperature down to ambient conditions after pretreatment completion. The heating system was a 4 kW model SBL-2D fluidized sand bath (Techne, Princeton, NJ, USA). After the pretreatment, the residual solids were washed with water to remove acid and other degradation compounds produced during the process.

IL pretreatment was performed at JBEI (Berkeley, CA, USA) using 1-ethyl-3-methylimidazolium acetate, abbreviated as [C2mim][OAc], at 140°C for 3 hours using 15% (wt/wt) loading of biomass to IL in a controlled temperature oil bath using a sealed stirred vessel. It took 30 minutes for the reactor to reach 140°C and 20 minutes to cool down to 60°C. The residual IL was removed and pretreated biomass material was recovered with a series of water and ethanol washes.

AFEX pretreatment was performed at the GLBRC (Biomass Conversion Research Laboratory, MSU, Lansing, MI, USA). The conditions were 140°C for 15 minutes at 60% (wt/wt) moisture with 1:1 anhydrous ammonia to biomass loading in a bench-top stainless steel batch reactor (Parr Instruments Company) [33]. It took 30 minutes for the reactor to reach 140°C and the ammonia was rapidly released, which immediately brought the biomass to room temperature. After the treatment, ammonia was removed by evaporation, leaving an essentially dry material. Hence AFEX is a dry to dry process, while IL and DA are dry to wet processes, as noted above.

### Compositional analysis

Extractive-based compositional analyses of the samples were performed according to the NREL LAPs: *Preparation of Samples for Compositional Analysis* (NREL/TP-510-42620) [42] and *Determination of Structural Carbohydrates and Lignin in Biomass* (NREL/TP-510-42618) [43]. The biomass was extracted with water and ethanol prior to the acid hydrolysis step. The sugar concentrations of the extracts were included in the composition.

### Microplate-based saccharification of pretreated biomass

Ternary enzyme mixture optimization assays were performed on AFEX, freeze-dried DA and freeze-dried IL pretreated corn stover samples at 0.2% glucan loading in a 96-well microplate as described by Gao *et al.*[44]. The assay was carried out at 15 mg protein/g glucan loading using three commercial enzyme mixtures (CTec2, HTec2 and Multifect Pectinase) at different ratios. The pH was maintained at 4.8 with 50 mM citrate buffer. The microplates were incubated at 50°C for 24 hours at 250 rpm. After hydrolysis, monomeric glucose and xylose concentrations of the liquid samples were determined colorimetrically using enzymatic assay kits, as described previously by Gao *et al.*[44].

### High solid loading hydrolysis

The washed (when used) solid streams from the three different pretreatments were hydrolyzed at 6% glucan loading in a fermenter equipped with a pitched blade impeller. The previously determined optimum enzyme mixtures were used for hydrolysis of the respective pretreated feedstocks. Hydrolysis was performed over a period of 3 days with 30 mg protein/g glucan enzyme loading at 50°C and 1,000 rpm. Samples were taken every 24 hours and the sugar concentrations were measured by HPLC. After 3 days of hydrolysis, the overall mass balances for the pretreated solids were determined as described previously by Garlock *et al.*[6].

### Fermentation

The sterile filtered hydrolysates resulting from DA, IL and AFEX treated corn stover at 6% glucan loading were fermented using a recombinant *S. cerevisiae* 424A (LNH-ST) strain capable of metabolizing both glucose and xylose to ethanol. Fermentations were carried out in 125 mL baffled shake flasks at 50 mL reaction volume. The experiments were initiated with an initial cell optical density (OD) of 2 measured at 600 nm, with and without nutrient supplementation (yeast extract (5 g/L) and tryptone (10 g/L)). Samples were taken at 4, 8, 12, 18, 24, 48, 72, 96 and 120 hours and their glucose, xylose and ethanol concentrations were determined by HPLC.

### Analytical methods

Monomeric sugars were quantified using a Shimadzu HPLC system equipped with an Aminex HPX-87P carbohydrate analysis column maintained at 60°C (Bio-Rad, Hercules, CA, USA) and Shimadzu refractive index detector (RID). Degassed HPLC grade water was used as a mobile phase at 0.6 mL/min. Injection volume was 10 μL with a run length of 20 minutes. Fermentation samples were analyzed for ethanol and residual sugars with the above mentioned HPLC system equipped with an Aminex HPX-87H column maintained at 50°C. Sulfuric acid (5 mM) was used as an eluent at 0.6 mL/min. Injection volume and run length was similar to HPX-87P column.

### Oligosaccharide analysis

Oligomeric sugar analysis was conducted on the hydrolysate liquid streams using an autoclave-based acid hydrolysis method at a 2 mL scale. Hydrolysate samples were mixed with 69.7 μL of 72% sulfuric acid in 10 mL screw-cap culture tubes and incubated in a 121°C bench-top hot plate for 1 hour, cooled on ice and filtered into HPLC vials. The concentration of oligomeric sugar was determined by subtracting the monomeric sugar concentration of the non-hydrolyzed samples from the total sugar concentration of the acid hydrolyzed samples. Sugar degradation was accounted for by running the appropriate sugar recovery standards along with the samples during acid hydrolysis.

## Abbreviations

[C2mim][OAc]: 1-Ethyl-3-methylimidazolium acetate; AFEX: Ammonia fiber expansion; ARP: Ammonia recycled percolation; BESC: BioEnergy Science Center; CAFI: Consortium for Applied Fundamentals and Innovation; CrI: Crystallinity index; CS: Corn stover; DA: Dilute acid; DOE: Department of Energy; GLBRC: Great Lakes Bioenergy Research Center; HPLC: High performance liquid chromatography; IL: Ionic liquid; JBEI: Joint BioEnergy Institute; LAP: Laboratory analytical procedure; LCC: Lignin-carbohydrate complex; MSU: Michigan State University; NREL: National Renewable Energy Laboratory; OD: Optical density; RID: Refractive index detector; SAA: Soaking in aqueous ammonia; SHF: Separate hydrolysis and fermentation; YEP: Yeast Extract Peptone.

## Competing interests

The authors declare no competing interests.

## Authors’ contributions

NU designed and executed all experiments. LDS and SPSC guided the design of experiments and data analysis. XY undertook preliminary composition analysis. BD initiated the collaboration and reviewed the manuscript. BS and SS prepared IL pretreated biomass and provided IL pretreatment-related data. XG, CW and RK prepared DA pretreated biomass and provided all DA pretreatment-related data. VB led and coordinated the overall project. NU, VB and LDS wrote the manuscript. All authors read, edited and approved the final manuscript.
